# Variability in home blood pressure and its association with renal function and pulse pressure in patients with treated hypertension in primary care

**DOI:** 10.1038/s41371-023-00874-2

**Published:** 2023-11-15

**Authors:** Ulrika Andersson, Peter M. Nilsson, Karin Kjellgren, Katie Harris, John Chalmers, Mikael Ekholm, Patrik Midlöv

**Affiliations:** 1 Center for Primary Health Care Research, Department of Clinical Sciences Malmö, Lund University, Malmö, Sweden; 2 Department of Clinical Sciences Malmö, Lund University, Malmö, Sweden; 3 University of Gothenburg Centre for Person-Centred Care (GPCC), Sahlgrenska Academy University of Gothenburg, Gothenburg, Sweden; 4 Department of Health, Medicine and Caring Sciences, Linköping University, Linköping, Sweden; 5 The George Institute for Global Health, University of New South Wales, Sydney, NSW Australia; 6 Department of Clinical Sciences, Danderyd Hospital, Karolinska Institute, Stockholm, Sweden

**Keywords:** Risk factors, Clinical trials, Hypertension

## Abstract

Blood pressure variability (BPV) represents a cardiovascular risk factor, regardless of mean level of blood pressure (BP). In this post-hoc analysis from the PERson-centredness in Hypertension management using Information Technology (PERHIT) study, we aimed to explore BPV in daily home measurements in hypertensive patients from primary care, to identify factors associated with high BPV and to investigate whether estimated glomerular filtration rate (eGFR) and pulse pressure, as markers of target organ damage (TOD), are associated with BPV. For eight consecutive weeks, 454 participants reported their daily BP and heart rate in their mobile phone, along with reports of lifestyle and hypertension-related factors. Systolic BP (SBP) values were used to calculate BPV with coefficient of variation (CV) as primary estimate. Background characteristics and self-reports were tested between fifths of CV in a linear regression model, adjusted for age and sex. Associations between BPV and eGFR and pulse pressure were tested with linear and logistic regression models. Higher home BPV was associated with higher age, BP, heart rate, and smoking. BPV was lower for participants with low alcohol consumption and treatment with calcium channel blockers. There was a significant association between BPV and pulse pressure (*P* = 0.015), and between BPV and eGFR (*P* = 0.049). Participants with high BPV reported more dizziness and palpitations. In conclusion, pulse pressure and eGFR were significantly associated with home BPV. Older age, high BP, heart rate, and smoking were associated with high BPV, but treatment with calcium channel blockers and low alcohol consumption was associated with low BPV. Trial registration: The study was registered with ClinicalTrials.gov [NCT03554382].

## Introduction

### Background

Blood pressure variability (BPV) is a significant prognostic marker for cardiovascular risk, independent of mean level of BP [1]. In previous guidelines, BPV was considered as a research area, with so far, little implication for clinical practice [2]. However, BPV research has progressed, and in a recently published position paper by Parati et al., consideration of BPV in clinical practice is recommended [1]. For long-term, or visit-to-visit BPV, there is strong evidence that it is related to cardiovascular morbidity and mortality [3]. Although there is limited data for mid-term, or day-to-day BPV, there is evidence of associations with cardiovascular events and mortality [4]. Mid-term or day-to-day BPV is preferably measured at home by the patient. Home BP monitoring has several benefits; it is easily accessible, and well-tolerated by patients [5]. Home BP monitoring also requires information and interaction with patients, thus supporting self-management of hypertension [2]. There are several studies describing home BPV, but most studies include few measurements, and there are no studies describing HBPV for a longer period than one month [6].

Previous studies describing BPV and lifestyle factors have found associations between increased BPV and excessive alcohol intake, smoking and sedentary lifestyle, among other factors [7, 8]. Increased day-to-day BPV is also associated with advanced age and is suggested as an independent marker of the aging process, as BPV shares the same pathophysiological changes as the hallmarks of aging [9]. There is a close relationship between home BPV and arterial stiffness, indicating that it may represent two sides of the same coin [9, 10]. Arterial stiffness is associated with target organ damage (TOD) and there is also evidence of an association between BPV and TOD in hypertensive patients [11, 12]. Estimated glomerular filtration rate (eGFR) is a marker for renal function and increased visit-to-visit variability of systolic BP (SBP) is associated with renal impairment in patients with hypertension [13]. Increased day-to-day variation of SBP has been associated with low eGFR in the general population [14] and in patients with diabetes [15]. Pulse pressure, the difference between systolic and diastolic BP, is a functional TOD and reflects the stiffening of large arteries [2, 16].

We have previously conducted a randomised controlled study (PERson-centredness in Hypertension management using Information Technology (PERHIT)) aimed at exploring the effect using an interactive web-based system for self-management of hypertension, including patients with treatment for hypertension in primary care [17]. The intervention included daily home monitoring of BP for eight consecutive weeks, thus providing an opportunity to explore day-to-day variability in home BP in patients with hypertension and to identify factors associated with increased BPV.

We hypothesize that increased home BPV reflecting arterial stiffness is associated with eGFR and pulse pressure as markers of TOD in patients with hypertension. Pulse pressure and eGFR were chosen as markers of TOD as they are commonly used and were accessible in our material.

### Objectives

The primary aim of this observational study was to explore BPV based on daily home measurements in hypertensive patients from primary care, and to identify factors associated with increased BPV. Further, we aimed to investigate whether eGFR and pulse pressure, as markers of TOD, were associated with day-to-day BPV, using the PERHIT study.

## Methods

### Study design and participants

The current study is a post-hoc, secondary analysis of data from the PERHIT study. In the PERHIT trial, the effect of using an interactive web-based system for self-management of hypertension in primary health care was evaluated [17]. In total, 949 patients with hypertension were included. Details of the trial have previously been published [18, 19]. In brief, the trial was conducted at 31 primary health care centres in southern Sweden. The patients were informed about the study by their nurse or physician, or through posters in the waiting areas at the primary health care centre. If eligible and willing to participate, the patients were randomised 1:1 to the intervention or control group, respectively. Inclusion criteria were Swedish-speaking adult patients with a diagnosis of hypertension and treatment with at least one antihypertensive drug, regardless of BP level. The participants took part in a baseline clinical assessment and returned for a follow-up visit after eight weeks and 12 months. Baseline and follow-up visits included measurement of BP (mmHg), heart rate (beats/min), height (m), weight (kg), and blood tests (total cholesterol, creatinine, HbA1c and cystatin C). The participants also filled out different questionnaires about demographic details, medication beliefs and adherence, participation in care, quality of life, and self-efficacy.

The participants in the intervention group of the PERHIT study received a BP monitor and installed a program called CQ (developed by Circadian Questions AB, Sweden) on their mobile phone. For eight consecutive weeks, patients in the intervention group measured their BP and heart rate every evening and reported their values in their mobile phone. They also reported lifestyle factors such as physical activity, stress, well-being, and hypertension-related factors, such as medication intake, side effects, and symptoms. The reported values were stored in a secure database, and by logging into a webpage the participants could get visual feedback of their reported values as graphs. The participating primary care physicians and nurses were encouraged to view and discuss the visual feedback together with the patient at the follow-up visit after eight weeks.

### Blood pressure measurement

The office BP was measured by the patient’s nurse or physician during the visits to the primary health care centre. The involved professionals had been instructed by the research team to ensure a standardized measurement technique. The office BP was measured with the patient in a sitting position and the mean of three consecutive measurements (mmHg) was recorded. A validated BP monitor (Microlife BP A6 BT, Switzerland) was used, and the patients were given the same type of monitor for home use during the intervention period.

The participants were instructed on BP measurement techniques to apply at home and had access to supporting instructive video films on the study web page.

### Blood pressure variability

There is no consensus regarding the preferred index for day-to-day BPV, but since BPV is strongly related to average BP level, it is recommended to incorporate mean BP [1, 5]. Standard deviation (SD) is commonly used, though it does not adjust for mean BP, thus is not recommend as a sole index. Coefficient of variation (CV) is the extent or variability in relation to the mean, expressed as SD/mean * 100. It is easily calculated and adjusts to some extent for mean BP level, but there can still be a correlation. To minimize correlation with mean BP, variation independent of the mean (VIM) was created. VIM is calculated with the formula SD/mean^x^, where x is obtained by a fitting curve through a plot of SD against mean using the model SD = a*mean^X^ [20]. VIM is impractical to use in a clinical setting since it is derived from the distribution of BP in the given population and cannot be compared across populations. Average real variability (ARV) is the average absolute difference between consecutive BP readings and has the advantage of considering the sequential order of BP readings [21].

### Statistical analysis

In this study, CV of home systolic BP was chosen as the primary parameter. Results for SD, VIM and ARV are also reported.

Participants were stratified according to fifths of CV of home systolic BP. Continuous variables are presented as means with standard deviations, and categorical variables as number and percentage.

We tested for a linear trend in the baseline characteristics by fitting the baseline characteristics as the dependent variable in a linear regression for continuous variables or a logistic regression for categorical variables, with the fifths of BPV as a continuous independent variable. The trend analysis was adjusted for age and sex.

Self-reported variables including lifestyle factors and symptoms from the CQ system are presented as the mean (SD) of the individuals mean of up to 57 consecutive readings. As for the baseline data, the self-reported variables were tested for a linear trend by fitting the self-reported variables as the dependent variable in a linear regression for continuous variables, or a logistic regression for categorical variables with the fifths of BPV as a continuous independent variable. The analysis was also adjusted for age and sex.

Pulse pressure and eGFR were used as indicators of TOD. Estimated glomerular filtration rate was calculated from baseline creatinine and cystatin C using the CAPA and LMrev equations, and the average value of the two equations was used as eGFR [22]. The LMrev and CAPA equations were chosen as they are developed in a Swedish population and have been shown to outperform other GFR estimations in similar settings [23]. Pulse pressure was calculated by subtracting the patients´ daily reported diastolic BP from the daily reported systolic BP. To test if there was a significant association between BPV and pulse pressure and eGFR, a linear regression analysis was conducted with the CV of SBP used as a continuous dependent variable and the pulse pressure and eGFR as independent variables, respectively. The model was adjusted for age, sex, smoking, BMI, cholesterol level, HbA1c, reported alcohol consumption at baseline and mean physical activity. The linear regression model for eGFR was also adjusted for baseline SBP. As pulse pressure is derived from SBP and DBP, the model for pulse pressure was not adjusted for SBP.

The association of CV of SBP and pulse pressure was also analysed in subgroups of participants based on antihypertensive treatment, with CV of SBP as a dependent variable and pulse pressure as an independent variable, adjusted for age, sex, smoking, BMI, cholesterol level, HbA1c, reported alcohol consumption at baseline and mean physical activity.

Odds ratios for high pulse pressure and low eGFR by fifths of CV were calculated using logistic regression model. High pulse pressure was defined as greater than or equal to 60 mmHg^2^, and low eGFR as less than 60 mL/min/1.73 m^2^. Statistical significance was tested with a logistic regression model adjusted for age, sex, smoking, BMI, cholesterol level, HbA1c, a reported alcohol consumption at baseline and mean physical activity and baseline SBP (only eGFR). Statistical significance was indicated by *P* < 0.05.

All statistical analyses were done in R version 4.1.2 and RStudio version 2022.2.2.485 (R Core Team (2021). R: A language and environment for statistical computing. R Foundation for Statistical Computing, Vienna, Austria. URL https://www.R-project.org/. RStudio Team (2022). RStudio: Integrated Development Environment for R. RStudio, PBC, Boston, MA URL http://www.rstudio.com/).

## Results

### Characteristics of study population

In total, 482 participants were randomised to the intervention group of the PERHIT trial. The maximum number of daily reports in the CQ system was 57 (the first day was numbered 0). Twenty-eight participants had ten or less daily reports in CQ and were not included in the analysis of BPV in this paper. Thus, 454 participants were included in the analysis.

The baseline characteristics overall and by fifths of CV of SBP are presented in Table 1. The mean age of participants was 62.5 years. There was majority of men (59%) and almost two thirds of the participants had a BP ≥ 140/90 mmHg at baseline. The mean duration of hypertension was 9.6 years, and the average number of antihypertensive drugs was 1.5. When comparing fifths of CV of SBP, the participants with the highest CV were more likely to be older, having a higher office SBP and DBP, and to be current smokers. The participants with the lowest CV of SBP were more likely to be on treatment with calcium channel blockers and drinking less than 1 unit of alcohol per week.

**Table 1:** Baseline characteristics of participants overall and according to fifths of CV of home systolic BP.

	Overall	CV of home SBP, %					
		1st fifth 2.4–5.4	2nd fifth 5.4–6.3	3rd fifth 6.3–7.2	4th fifth 7.2–8.4	5th fifth 8.4–15.5	*P*-value
*N*	454	91	91	90	91	91	
Age (years), SD and range	62.5 ± 9.8 25–85	59.2 ± 10.6 30–77	62.0 ± 9.9 31–80	63.0 ± 9.3 25–82	63.7 ± 9.3 33–83	64.4 ± 8.6 43-85	<0.001
Sex, women, *n* (%)	187 (41.2)	29 (31.9)	41 (45.1)	45 (50.0)	40 (44.0)	32 (35.2)	0.850
Proportion with BP < 140/90 mmHg, *n* (%)	165 (36.3)	38 (30.8)	43 (47.3)	33 (36.7)	20 (22.0)	31 (34.1)	<0.001
Office SBP (mmHg), mean and SD	143.7 ± 16.2	139.3 ± 12.3	140.7 ± 15.0	143.0 ± 16.3	150.1 ± 17.5	145.3 ± 17.4	<0.001
Office DBP (mmHg), mean and SD	84.7 ± 8.9	84.5 ± 8.3	84.3 ± 9.6	83.6 ± 8.4	85.8 ± 9.1	85.2 ± 9.2	0.008
Heart rate (beats/minute), mean and SD	72.2 ± 12.1	71.9 ± 12.0	71.4 ± 12.2	72.8 ± 13.5	71.8 ± 12.3	72.9 ± 10.7	0.319
BMI (kg/m^2^), mean, SD and range	28.8 ± 4.4 18–45	29.0 ± 4.8 19–45	28.2 ± 3.9 20–40	28.3 ± 4.0 20–42	29.4 ± 4.4, 20–44	29.1 ± 4.7 18–42	0.061
HbA1c (mmol/mol), mean and SD	38.8 ± 7.5	38.8 ± 7.3	38.0 ± 6.2	38.2 ± 6.1	39.5 ± 8.3	39.4 ± 9.0	0.736
eGFR (mL/min/1.73 m^2^), mean and SD	75.9 ± 13.7	78.3 ± 14.5	76.1 ± 14.1	76.8 ± 13.9	76.0 ± 13.1	72.1 ± 12.6	0.466
Total cholesterol (mmol/L), mean and SD	5.0 ± 1.0	5.0 ± 1.1	4.9 ± 1.0	5.1 ± 1.0	4.9 ± 0.9	5.0 ± 1.0	0.877
Hypertension (years), mean and SD	9.6 ± 8.8	10.1 ± 9.1	9.9 ± 9.1	8.7 ± 7.2	9.7 ± 9.2	9.6 ± 9.4	0.051
Antihypertensive drugs, mean and SD	1.5 ± 0.8	1.5 ± 0.8	1.5 ± 0.8	1.6 ± 0.8	1.4 ± 0.8	1.6 ± 0.9	0.621
Diuretics, *n* (%)	63 (13.9)	10 (11.0)	11 (12.1)	12 (13.3)	14 (15.4)	16 (17.6)	0.334
Beta-blockers, *n* (%)	108 (23.8)	20 (22.0)	25 (27.5)	22 (24.4)	19 (20.9)	22 (24.2)	0.383
Ca-antagonists, *n* (%)	160 (35.2)	41 (45.1)	29 (31.9)	35 (38.9)	29 (31.9)	26 (28.6)	0.024
RAS blockers, *n* (%)	349 (76.9)	66 (72.6)	68 (75.8)	71 (77.8)	66 (72.5)	78 (85.7)	0.108
Other antihypertensive drugs *n* (%)	3 (0.7)	1 (1.1)	0	0	0	2 (2.2)	0.580
Diabetes, *n* (%)	57 (12.6)	14 (15.4)	10 (11.0)	7 (7.8)	10 (11.0)	16 (17.6)	0.913
Heart failure, *n* (%)	9/432 (2.1)	2/91 (2.2)	1/91 (1.1)	1/89 (1.1)	2/91 (2.2)	3/91 (3.3)	0.690
Current smoker, *n* (%)	23/447 (5.1)	2/89 (2.2)	3/89 (3.4)	4/90 (4.4)	5/91 (5.5)	9/88 (10.2)	0.013
Previous smoker, *n* (%)	115/447 (25.7)	20/89 (22.5)	20/89 (22.5)	22/90 (24.4)	26/91 (28.6)	27/88 (30.7)	0.294
Alcohol <1 standard drinks per week, *n* (%)	166/444 (37.4)	38/89 (42.7)	42/88 (47.7)	32/90 (35.6)	26/89 (29.2)	28/88 (31.8)	0.006
Alcohol 1–9 standard drinks per week, *n* (%)	251/444 (56.6)	47/89 (52.8)	44/88 (50.0)	51/90 (56.7)	57/89 (64.0)	52/88 (59.1)	0.092
Alcohol ≥10 or standard drinks per week, *n* (%)	27/444 (6.1)	4/89 (4.5)	2/88 (2.3)	7/90 (7.8)	6/89 (6.7)	8/88 (9.1)	0.056

A linear trend in the baseline characteristics was tested for by fitting the baseline characteristics as the dependent variable in a linear regression for continuous variables or a logistic regression for categorical variables, with the fifths of blood pressure variability as a continuous independent variable.

*CV* coefficient of variation, *SD* standard deviation, *SBP* systolic blood pressure, *DBP* diastolic blood pressure.

There was no correlation between CV of SBP and marital status, educational level, or occupation for the participants.

### BP levels and BPV

The mean office SBP at baseline was 143.7 ± 16.2 mmHg and the DBP was 84.7 ± 8.9 mmHg for the study participants. After eight weeks the mean office SBP was 139.9 ± 15.9 mmHg and the DBP 83.6 ± 9.4 mmHg. In all, 49.1% of the participants had a BP < 140/90 mmHg after eight weeks. Across the eight weeks of home measurement, mean SBP was 135.6 ± 11.0 mmHg and mean DBP was 79.1 ± 7.3 mmHg, respectively. The mean home measurements were significantly lower than the mean office measurements at 8 weeks (*P* < 0.001).

The mean values of different BPV indices of home SBP are presented in Table 2. There was no significant difference in BPV between men and women for any index.

**Table 2:** Mean values of different BPV indices of home systolic and diastolic BP.

BPV index	Home SBP	Home DBP
CV (%), mean and SD	7.0 ± 2.0	7.7 ± 2.9
SD, mean and SD	9.6 ± 2.9	6.1 ± 2.3
VIM, mean and SD	9.5 ± 2.9	3.0 ± 1.1
ARV, mean and SD	7.5 ± 2.8	4.6 ± 1.7

*BPV* blood pressure variability, *CV* coefficient of variation, *SD* standard deviation, *SBP* systolic blood pressure, *DBP* diastolic blood pressure, *VIM* variation independent of the mean, *ARV* average real variability.

### Self-reported variables in fifths of CV of SBP

The median number of daily reports was 53 (interquartile range 47–55). In some instances, the participants answered one or more questions but did not report a BP value. The median number of daily reports of SBP was 52 (interquartile range 46–55).

When comparing fifths of CV of SBP and self-reported variables, the participants with the highest BPV had a higher BP, higher heart rate, and higher pulse pressure. They also reported more dizziness and palpitations (Table 3).

**Table 3:** The participants’ self-reported data (mean of individual means) and fifths of CV for home systolic BP.

	CV of home SBP					
CQ variables	1st fifth	2nd fifth	3rd fifth	4th fifth	5th fifth	*P*-value
HSBP (mmHg), mean and SD	132.9 ± 8.3	132.5 ± 9.5	135.6 ± 10.9	138.4 ± 11.6	138.8 ± 12.8	<0.001
HDBP (mmHg), mean and SD	79.3 ± 6.5	78.0 ± 6.5	78.4 ± 7.6	79.6 ± 7.4	80.0 ± 8.2	0.001
Pulse pressure (mmHg), mean and SD*	53.7 ± 8.4	54.8 ± 8.8	57.3 ± 9.0	58.9 ± 10.2	58.8 ± 10.6	<0.001
Heart rate (beats/min), mean and SD	70.3 ± 9.1	70.2 ± 9.5	71.1 ± 9.6	72.4 ± 9.5	74.1 ± 9.6	<0.001
Medication intake, mean and SD 1 = taken, 0 = not taken	1.0 ± 0.1	1.0 ± 0.1	1.0 ± 0.1	1.0 ± 0.1	1.0 ± 0.1	0.775
Physical inactivity, mean and SD 1 = not at all, 5 = very much	3.5 ± 0.6	3.3 ± 0.6	3.4 ± 0.6	3.3 ± 0.5	3.3 ± 0.6	0.288
Stress, mean and SD 1 = very much, 5 = not at all	4.6 ± 0.4	4.6 ± 0.4	4.6 ± 0.5	4.6 ± 0.4	4.5 ± 0.5	0.059
Sleep, mean and SD 1 = very bad, 5 = very good	3.6 ± 0.5	3.7 ± 0.6	3.6 ± 0.6	3.6 ± 0.6	3.7 ± 0.6	0.506
General health, mean and SD 1 = very bad, 5 = very good	3.9 ± 0.5	4.0 ± 0.5	3.9 ± 0.5	3.9 ± 0.5	3.9 ± 0.5	0.139
Tiredness, mean and SD 1 = very much, 5 = not at all	4.1 ± 0.6	4.2 ± 0.5	4.0 ± 0.7	4.2 ± 0.6	4.1 ± 0.6	0.396
Dizziness, mean and SD 1 = not at all, 5 = very much	1.1 ± 0.2	1.1 ± 0.2	1.2 ± 0.3	1.2 ± 0.3	1.2 ± 0.4	0.023
Headache, mean and SD 1 = not at all, 5 = very much	1.3 ± 0.4	1.3 ± 0.4	1.4 ± 0.4	1.3 ± 0.4	1.3 ± 0.3	0.299
Palpitations, mean (SD) 1 = not at all, 5 = very much	1.1 ± 0.3	1.1 ± 0.2	1.2 ± 0.3	1.2 ± 0.3	1.2 ± 0.4	0.018
Restlessness, mean and SD 1 = not at all, 5 = very much	1.2 ± 0.3	1.2 ± 0.3	1.2 ± 0.4	1.3 ± 0.4	1.3 ± 0.4	0.091

*CV* coefficient of variation, *SD* standard deviation, *SBP* systolic blood pressure, *HSBP* home systolic blood pressure, *HDBP* home diastolic blood pressure.

*Pulse pressure was calculated from the participants reports of SBP and DBP. A linear trend in the self-reported values was tested for by fitting the self-reports as the dependent variable in a linear regression, with the fifths of BPV as a continuous independent variable, adjusted for sex and age.

### Association between BPV and eGFR and pulse pressure

**Fig. 1:**
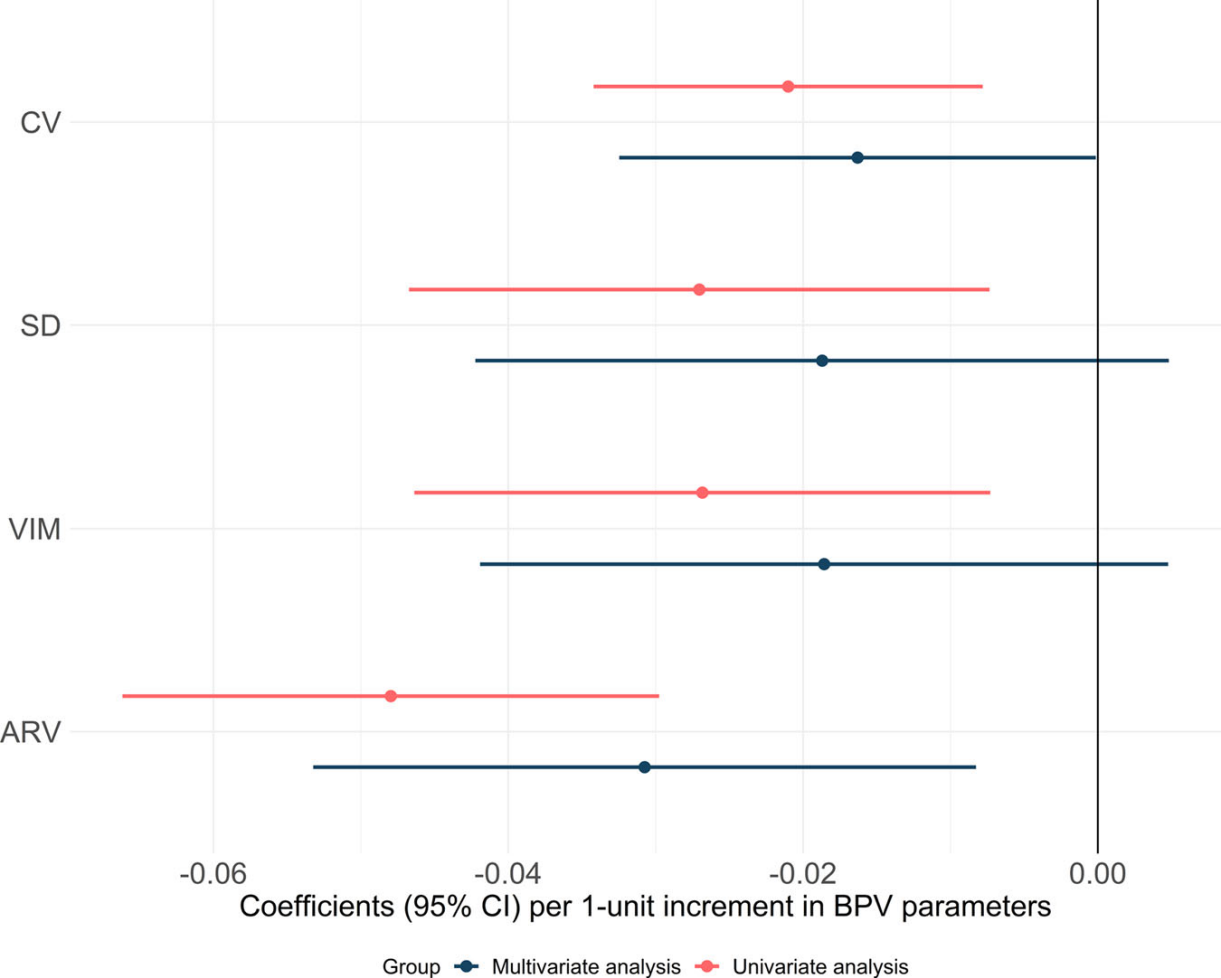
Linear regression analysis of the relationship between different parameters for BPV and eGFR. Multivariable analysis included age, sex, smoking, baseline SBP, BMI, cholesterol level, HbA1c, reported alcohol consumption at baseline and mean physical activity as independent variables. BPV blood pressure variability, CV coefficient of variation, SD standard deviation, SBP, VIM variation independent of the mean, ARV average real variability.

In the unadjusted analysis, higher CV of SBP was significantly associated with lower eGFR (*P* = 0.002). After adjusting for age, sex, smoking, baseline SBP, BMI, cholesterol level, HbA1c, reported alcohol consumption at baseline and mean physical activity the association was still significant (*P* = 0.049). When using SD and VIM as measurements for BPV, the multivariate analysis did not show significant associations. When using ARV as measurement, the association was significant after adjustments (Fig. 1). The results were similar when using the 2021 CKD-EPI equation instead of the LMrev and CAPA equations for eGFR estimation (see Supplementary Material).

**Fig. 2:**
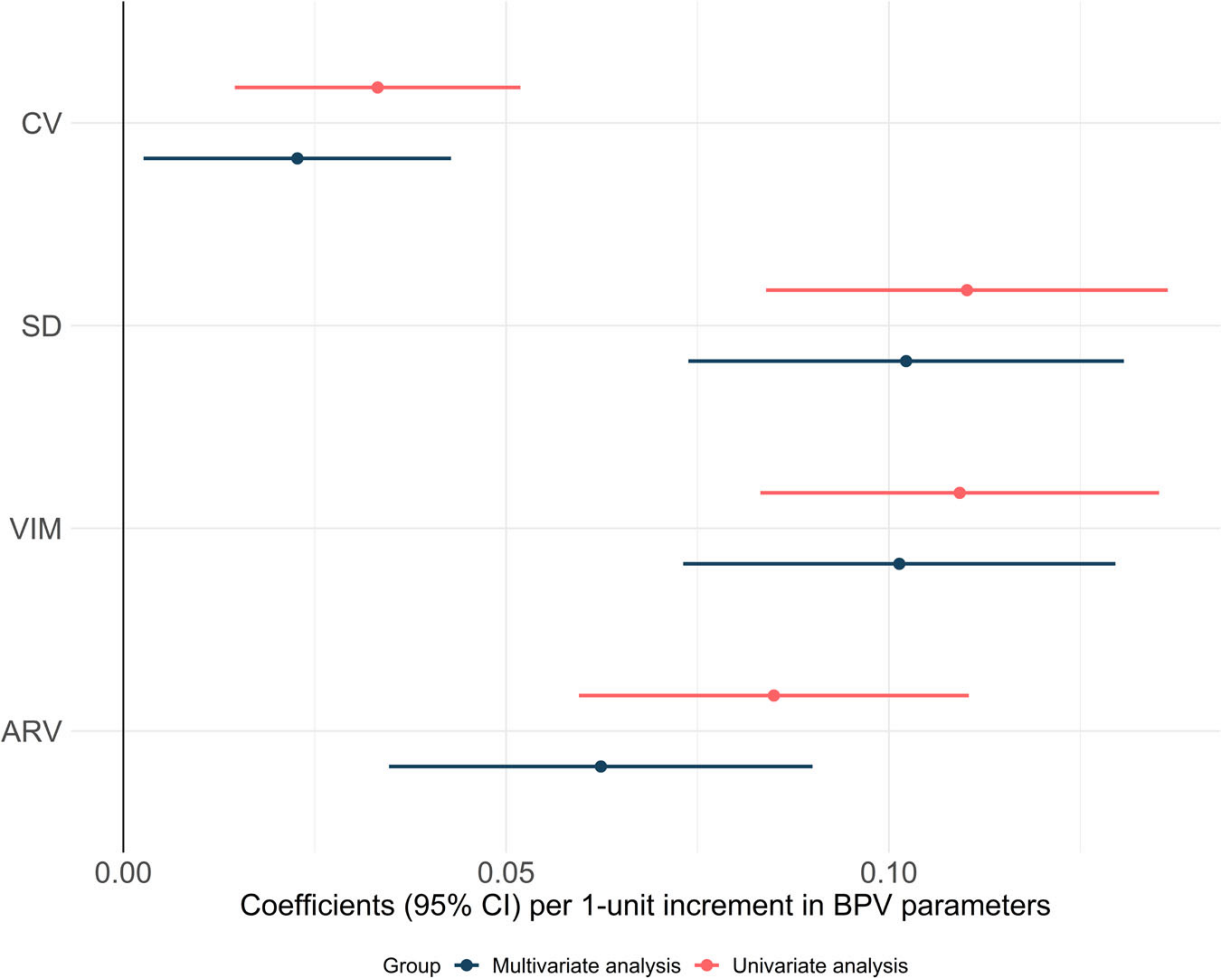
Linear regression analysis of the relationship between different parameters for BPV and pulse pressure. Multivariable analysis included age, sex, smoking, BMI, cholesterol level, HbA1c, reported alcohol consumption at baseline and mean physical activity as independent variables. BPV blood pressure variability, CV coefficient of variation, SD standard deviation, SBP, VIM variation independent of the mean, ARV average real variability.

Higher CV of SBP was significantly associated with higher pulse pressure in the unadjusted analysis (*P* < 0.001) as well as after adjusting for age, sex, smoking, BMI, cholesterol level, HbA1c, reported alcohol consumption at baseline and mean physical activity (*P* = 0.027) (Fig. 2). When adjusting for baseline SBP or mean arterial pressure, the association between CV of SBP and pulse pressure became non-statistically significant (*P* = 0.23 and 0.30 respectively), but for the other estimates of BPV the association remained significant.

There was no significant association between CV of SBP and pulse pressure in the group of participants treated with diuretics (*n* = 63), beta-blockers (*n* = 108) or RAS-inhibitors (*n* = 349). However, there was a significant association between CV of SBP and pulse pressure in the group treated with calcium channel blockers (*n* = 160) (Table 4).

**Table 4:** Linear regression analysis for groups with different antihypertensive treatment at baseline.

CV for SBP in groups based on antihypertensive treatment	Pulse pressure			
	β	SE	*P*-value	95% CI
Diuretics (*n* = 63)	−0.0001	0.030	0.996	−0.061–0.061
Beta-blockers (*n* = 108)	0.019	0.021	0.364	−0.022–0.060
Calcium channel blockers (*n* = 160)	0.038	0.015	0.014	0.008–0.068
RAS blockers (*n* = 349)	0.018	0.012	0.129	−0.005–0.041

Note that participants may have been taking more than one antihypertensive treatment.

Adjusted for age, sex, smoking, BMI, cholesterol level, HbA1c, alcohol consumption and physical activity.

*CV* coefficient of variation, *SBP* systolic blood pressure, *SE* standard error.

### Logistic regression analysis for low eGFR and high pulse pressure in fifths of CV

A logistic regression analysis of CV for low eGFR and high pulse pressure is presented in Table 5. The multivariable analysis included age, sex, smoking, BMI, cholesterol level, HbA1c, reported alcohol consumption at baseline and mean physical activity and systolic BP (only for eGFR) as independent variables. The lowest fifth of CV was used as reference. There were no significant associations between CV for SBP fifths and low eGFR in the univariable analysis or in the multivariable analysis. In the univariable analysis for pulse pressure, the odds ratios for the three highest fifths of CV compared with the lowest fifth were significantly higher, and in the multivariable analysis the two highest fifths remained significantly higher.

**Table 5:** Logistic regression analysis for CV of SBP in fifths, when eGFR and pulse pressure are used as categorial variables.

Fifths of CV of SBP	Low eGFR		High pulse pressure	
	OR (95% CI)	*P*-value	OR (95% CI)	*P*-value
Univariable analysis				
1st fifth	Reference	Reference	Reference	Reference
2nd fifth	2.00 (0.78–5.56)	0.161	1.56 (0.78–3.19)	0.216
3rd fifth	1.20 (0.41–3.57)	0.736	2.18 (1.11–4.39)	0.026
4th fifth	1.65 (0.62–4.68)	0.324	3.41 (1.77–6.81)	<0.001
5th fifth	2.37 (0.95–6.49)	0.075	3.12 (1.62–6.23)	<0.001
Multivariable analysis				
1st fifth	Reference	Reference	Reference	Reference
2nd fifth	1.79 (0.58–5.82)	0.347	1.60 (0.75–3.47)	0.129
3rd fifth	0.98 (0.30–3.29)	0.952	1.88 (0.90–4.02)	0.072
4th fifth	1.23 (0.41–3.94)	0.544	2.84 (1.38–6.01)	0.002
5th fifth	2.11 (0.72–6.61)	0.146	2.50 (1.21–5.32)	0.005

eGFR <60 mL/min/1.73 m^2^ is defined as low and pulse pressure ≥60 is defined as high. The multivariable analyses are adjusted for age, sex, smoking, BMI, cholesterol level, HbA1c, alcohol use and physical activity. The multivariable analysis for eGFR is also adjusted for baseline SBP.

*CV* coefficient of variation, *eGFR* estimated glomerular filtration rate, *OR* odds ratio.

## Discussion

### Principal findings

In this observational study of 454 patients with hypertension treated in primary care, we analysed CV of home SBP, monitored for up to eight weeks, as a measurement for day-to-day BPV. Higher BPV was associated with higher age, higher BP and heart rate, and smoking. On the contrary, BPV was lower for the participants with a low alcohol consumption and those treated with calcium channel blockers. There was a significant association between higher BPV and higher pulse pressure, and between higher BPV and lower eGFR. The patients with highest BPV reported more dizziness and palpitations.

### Comparisons to previous work

The factors associated with BPV in our study were mostly consistent with previous literature. With older age comes decreased elasticity and compliance of the large arteries causing arterial stiffness. Increased BPV follows [9]. Studies focusing on elderly people report higher values of BPV and advanced age is consistently reported as a factor associated with increased BPV [7, 24].

Increased mean BP and smoking is also frequently reported to be associated with increased BPV [7]. Previous research in the general population has found an association between low heart rate and BPV [25]. In our study we found an opposite association; the participants with at higher BPV had a higher heart rate measured at home. The explanation may reflect different study populations, as there is an association between elevated heart rate and hypertension [26].

Female sex is often reported as associated with increased BPV [7]. In our population there was no significant difference in BPV between men and women. Gender aspects may vary between different ethnicities and most previous research in the area has been conducted in Asian populations. As for all studies involving BPV, results may also vary with the BP measurement method and the BPV index used.

There is evidence of an association between excessive alcohol consumption and increased arterial stiffness [27]. In our study, low BPV was associated with low alcohol intake, which is in line with previous findings. The association is not without controversy; some studies have found no correlation between alcohol intake and arterial stiffness. There are also results from several studies supporting a J-shaped relationship, indicating that moderate alcohol consumption is associated with lower arterial stiffness, but this may be the case only in persons with normal BP [27, 28].

Previous studies have reported differences in BPV depending on antihypertensive drug treatment [7]. Treatment with beta-receptor blockers is reported to be associated with higher BPV [29] but treatment with calcium channel blockers with lower BPV [25, 30]. Our findings suggest an association between treatment with calcium channel blockers and lower BPV, but the findings must be interpreted cautiously, as our data are purely observational and do not reflect a randomised drug treatment intervention. Further, combinations of different antihypertensive drugs were not studied. Current knowledge of the varying effect of different antihypertensive drugs on BPV is mainly based on *post-hoc* studies and has no implications yet in clinical practice [1]. Further research is needed to establish which antihypertensive drug class is more beneficial in patients with increased BPV.

In this study we could not demonstrate a significant association between participants reported physical activity and BPV. Lin et al. found no association between physical fitness and long-term BPV in young male adults [31] and Uusitalo et al. described similar findings in older men [32]. Maseli et al. described that a healthy lifestyle was associated with a lower BPV [33]. The lifestyle associated variables in our study – BMI, cholesterol and HbA1c – did not differ between fifths of CV. This may be explained by difference in populations, Maseli’s study participants consisted of young healthy adults. In our population of patients with treated hypertension, more homogenous results might be expected than in the general population.

We did find a significant association between BPV and eGFR in the multivariate linear regression model. Kubozono et al. [14] has described day-to-day BPV and the relationship to eGFR as a marker for kidney function in the general population when a significant association was found. There were differences in study populations, as Kubozono included participants from the general population where a wider distribution of eGFR would be expected. The participants in Kubozono’s study were also older and had a lower mean eGFR than found in the present study. Our results differed when using different indices for BPV. For CV and ARV for SBP, there was a significant association between BPV and eGFR after adjusting for age, sex, smoking, baseline SBP, BMI, cholesterol level, HbA1c, self-reported alcohol consumption at baseline, and mean physical activity, but not for the other indices of SBP. This indicates that the indices used to describe BPV are not interchangeable why further research is needed to establish which index should be preferred for home BPV. ARV is primarily considered appropriate for short-term BPV within 24 h for which the successive order of measurements is of greater significance [1].

Furthermore, pulse pressure was significantly associated with BPV. The relationship between arterial stiffness and pulse pressure is well explored [34, 35] and pulse pressure is a functional equivalent of a structural TOD, as expressed by Mancusi et al. [16]. Imai et al. reported that pulse pressure is a predictor of BPV in the general population [36]. Our findings suggest that this is also the case in patients with treated hypertension.

BPV is a research area with many unanswered questions. The results of previous studies have not given unanimous answers. The evidence supports an association between day-to-day BPV and markers of TOD in patients with hypertension, although the causation is not clearly established. Some factors associated with BPV are undisputed, such as old age and increased BP levels. The result for other factors varies in different populations, with different estimates of BPV, and with different techniques and timing for BP measurements. As reasoned by Boubouchariopoulou et al., different BPV measurement methods (such as office BP, home BP and ambulatory BP) represent different pathophysiological aspects and so far, the optimal combination of BP index and BP measurement method is unknown [24].

### Strengths and limitations

A strength of this study is the large number of values of home BP readings, extending over eight weeks. It was conducted in primary care, where most patients with hypertension are treated, and included a variety of patients with hypertension with respect to age and socioeconomic background. We had a low dropout rate, only 28 participants out of 482 were not included in our present analysis of BPV.

There are some important limitations of the study. First, the present study design was a *post-hoc*, non-randomised, observational analysis, as the intervention trial was not primarily designed for this purpose. Thus, the results need to be interpreted with some caution. Second, the study included only Swedish-speaking patients, as that was an inclusion criterion, and 95% of the patients were born in Sweden. It is possible that the results would be different if the study population had been more heterogenous regarding ethnicity. Third, we used eGFR and pulse pressure as markers of TOD and one might argue that there exist several other markers of TOD that could be analysed, such as albuminuria. Since our data is limited, it was not possible to include other markers of TOD. Finally, our analyses were based on BPV during eight weeks, and this might have been too short a time to identify factors associated with increased BPV.

In conclusion, this study demonstrated an association between variability of home BP and pulse pressure and between home BPV and eGFR, in treated hypertensive patients. Older age, high mean BP, increased heart rate, and smoking were associated with high BPV, but treatment with calcium channel blockers and low alcohol consumption was associated with low BPV.

## Summary

What is known about this topic

- Day-to-day BPV is associated with cardiovascular morbidity and mortality. It is unclear which index should be preferred to measure BPV.
- Previous research has described several factors associated with BPV.
- Home BPV and arterial stiffness are closely related. Arterial stiffness is associated with TOD and there may also be an association between BPV and TOD.

What this study adds

- In this study including patients with treated hypertension in primary care, high BPV was associated with old age, high mean BP, increased heart rate and smoking. Low alcohol consumption and treatment with calcium channel blockers was associated with low BPV.
- Home BPV was significantly associated with pulse pressure and eGFR, as markers of TOD.

### Supplementary information

Estimations of eGFR

## Data Availability

The data that support the findings of this study are available from the PERHIT-study, but restrictions apply to the availability of these data, which were used under license for the current study, and so are not publicly available. Data are however available from the authors upon reasonable request. R code for the regression analyses and calculations of BPV-indices are available from the author upon request.
