# Supplementary material for: Variability in home blood pressure and its association with renal function and pulse pressure in patients with treated hypertension in primary care
Source: J Hum Hypertens. 2023 Nov 15;38(3):212–20. doi: 10.1038/s41371-023-00874-2 (PMC10940151; doi:10.1038/s41371-023-00874-2)
Supplement: Supplementary file 1 — Estimations of eGFR [file 41371_2023_874_MOESM1_ESM.pdf]

Supplementary Material

Variability in home blood pressure and its association with renal function and pulse pressure in patients with treated hypertension in primary care

Estimations of eGFR – LMrev and CAPA versus CKD-EPI

Using 2021 CKD-EPI equation for eGFR estimation in our material, mean eGFR was 85.8 ±16.6 mL/min/1,73m^2, compared to 75.9 ±13.7 mL/min/1,73m^2 using LMrev and CAPA equations.

Linear analysis, with CV of SBP as dependent variable and eGFR (LMrev and CAPA versus CKD-EPI) as predictor.

	Coefficient	SE	P-value	95% CI
Unadjusted				
**LMrev and CAPA**	-0.021	0.007	0.002	-0.034 – -0.008
**CKD-EPI**	-0.017	0.006	0.002	-0.028 – -0.006
Adjusted for age, sex, smoking and SBP at baseline				
**LMrev and CAPA**	-0.013	0.008	0.119	-0.029 – 0.003
**CKD-EPI**	-0.011	0.007	0.098	-0.024 – 0.002
Adjusted for age, sex, smoking, SBP at baseline, BMI, total cholesterol, Hba1c, alcohol consumption and mean physical activity				
**LMrev and CAPA**	-0.016	0.008	0.049	-0.033 – -0.000
**CKD-EPI**	-0.014	0.007	0.033	-0.027 – -0.001

When adjusting for multiple variables, the result was significant using the LMrev and CAPA equations, as well as using the CKD-EPI equation.

When using SD and VIM of SBP as measurements for BPV, the results were similar using the different eGFR estimations, with a significant association in the unadjusted analysis but non-significant results following any level of adjustment. When using ARV of SBP as measurement, the association was significant even after adjustments for either of the eGFR equations.
